# Dr. Mordecai Price, Philadelphia

**Published:** 1905-01

**Authors:** 


### OBITUARY.

---

### Dr. Mordecai Price, Philadelphia.

The tragic death of this distinguished surgeon, brief notice of which was made in the Journal for December, 1904, occurred October 29, 1904. It appears that Dr. Price early in the previous evening made an operation for appendicitis, assisted by his son-in-law, Dr. J. C. Deal, and by Dr. W. K. Shea. Dr. Deal walked home with his father-in-law, remaining with him a few moments and then went to his own home, leaving Dr. Price apparently in good health. Early next morning the housemaid discovered his dead body lying on the floor of his office, still clad in his overcoat, his hand grasping an instrument case. It was apparent that he sat down in a chair and was seized with apoplexy, fell on the floor and soon afterward expired.

It is difficult to conceive of a nearer approach to an ideal death than that which befell Mordecai Price. After performing an operation of emergency to save the life of a fellow-being, the great surgeon walked into his office, sat down among his books and instruments and amidst their quiet environment breathed out his life.

Mordecai Price was born in Rockingham County, Va., in 1844. At the age of 14 years he lost a leg through a machine accident, which confined him in a hospital for 14 months, the greater portion of the time being spent in bed. During this time he became deft with the crochet needle, one of his creations commanding a remarkable price. While still a lad he came to Philadelphia, receiving his preliminary education at Kennett Square, Pa., and St. Edward's Institute, New York. Soon after completing his academic studies he entered the medical school of the University of Pennsylvania, from which he graduated in 1869.

Dr. Price began the practice of his profession at once in the city of Philadelphia and continued to be engaged therein without interruption, with a single exception, until his death. About six years ago he suffered an attack of typhoid fever with prolonged sequelæ, including suppurative pleuritis, for which he sustained resection of a rib, recovery being complete. This was the only break in his long continued and unusually successful practice.

Early in his professional career Dr. Price manifested a predilection for surgery and soon found himself entirely absorbed with surgical practice. Meanwhile, a younger brother, Dr. Joseph Price, having been his pupil, graduated in medicine and the two brothers became associated in the active practice of surgery, paying particular attention to the abdominal and pelvic cavities. It was an interesting picture to see these two brothers, always affectionately attached to each other, on either side of the operating table solving the most difficult problems of abdominal and pelvic surgery with scarcely a word spoken between them, each knowing the desire of the other, and each playing the part alternately of principal and assistant as became the circumstances. Of late years the Price brothers have been in close relationship in the management of one of the largest and most successfully conducted private hospitals in any of the Atlantic coast cities.

Dr. Price was twice married, first to Miss Fanny Remington of Philadelphia and, after her death, second to Miss Margaret Ryan of Baltimore. He is survived by three daughters, two of whom married physicians,—Dr. J. C. Beal and Dr. E. A. Shumway—and the third, Miss Lucy Price, remained at home as the head of his own household.

In his personality Dr. Price was a most lovable character; he carried the sunshine of a cheerful countenance and buoyant manner into every household where his ministrations led him; he was one of the masters of modern surgery which he practised with almost unexampled success, and he left a fame unspotted that will ever be cherished by his children and his children's children even to the fourth generation.

